# Complete mitochondrial genome of *Coscinodiscus granii* (Coscinodiscophyceae, Bacillariophyta)

**DOI:** 10.1080/23802359.2021.1951135

**Published:** 2021-07-15

**Authors:** Hailong Huang, Huiyin Song, Zengxia Zhao, Feng Liu, Nansheng Chen

**Affiliations:** a CAS Key Laboratory of Marine Ecology and Environmental Sciences, Institute of Oceanology, Chinese Academy of Sciences, Qingdao, China; b Laboratory of Marine Ecology and Environmental Science, Qingdao National Laboratory for Marine Science and Technology, Qingdao, China; c Center for Ocean Mega-Science, Chinese Academy of Sciences, Qingdao, China; d Department of Molecular Biology and Biochemistry, Simon Fraser University, Burnaby, Canada; e Jiaozhou Bay National Marine Ecosystem Research Station, Institute of Oceanology, Chinese Academy of Sciences, Qingdao, China

**Keywords:** Diatoms, mitochondrial genome, *Coscinodiscus granii*, Coscinodiscophyceae

## Abstract

*Coscinodiscus* is a genus common in marine phytoplankton, with some species thought to have a significant negative ecological impact. However, the availability of their genome sequences is rather limited. Here, we assembled and annotated the first complete mitochondrial genome (mtDNA) of the species *Coscinodiscus granii* L.F.Gough 1905, as part of our efforts to gain a better understanding of the genetic characteristics of *Coscinodiscus* taxa at a genomic level. The circular mtDNA was 34,970 bp in length and encoded 60 genes, including 32 protein-coding genes (PCGs), 24 transfer RNA (tRNA) genes, two ribosomal RNA (rRNA) genes, and two conserved open reading frames (*orf*s). The overall GC content of *C. granii* mtDNA was 24.30%, which was slightly lower than that of *C. wailesii* (25.00%), the first species in the genus *Coscinodiscus* whose mtDNA has been reported, and higher than that of *Melosira undulata* (21.60%), the first species in the class Coscinodiscophyceae whose mtDNA has been reported. As expected for congeneric species, phylogenetic analysis using concatenated amino acid sequences of 27 shared PCGs suggested that *C. granii* has a closer evolutionary relationship with *C. wailesii*. *Coscinodiscus* was found to be monophyletic in the phylogeny. The complete mtDNAs of more *Coscinodiscus* species will facilitate the exploration of the evolutionary relationships of species in the Class Coscinodiscophyceae.

*Coscinodiscus* is a species-rich genus among diatoms, with 174 taxonomically accepted species in AlgaeBase (Guiry and Guiry 2021), among which 49 species have been described in China (Chen et al. 2021; Chen and Chen 2021; Chen and Huang 2021; Chen and Zhang 2021). Some of these *Coscinodiscus* species have been found to form blooms that can cause serious damage to the aquaculture of macro rhodophyta (Nishikawa et al. 2010). Some *Coscinodiscus* species can be relatively large with valves up to 500 µm in diameter (Kühn and Raven 2008). The species *Coscinodiscus granii* L.F. Gough 1905 can be up to 300 µm in diameter (Boalch 1971; Yan 2017) and has a worldwide distribution. *Coscinodiscus granii* has been reported to form characteristic autumn blooms in the Baltic Sea and presented throughout the year in ‘low to moderate’ abundance in the North European Seas (Wasmund et al. 2003; Kraberg et al. 2010). *Coscinodiscus granii* poses an impact on the whole carbon pool and plays a role in the marine ecosystem, due to its relatively large cell sizes and high carbon content (Zhang et al. 2007).

The strain CNS00554 analyzed in this study was isolated in water samples collected during an expedition to the Jiaozhou Bay, China (36°08.031’N, 120°11.309’E) in August 2020 onboard the research vehicle ‘Innovation,’ using same sampling protocol as previously reported in Wang et al. (2021). The strain CNS00554 was annotated as *C. granii* based on its morphological features (Hasle and Lange 1992; Goessling et al. 2016) (Figure 1(B,C)) and molecular characterization based on common molecular markers including full-length 18S rDNA (Damsté et al. 2004) and *rbcL* (Theriot et al. 2010) (Figure S1). The *C. granii* culture is maintained in the collection of marine algae in KLMEES of IOCAS (Nansheng Chen, chenn@qdio.ac.cn) under the voucher number CNS00554.

**Figure 1.**
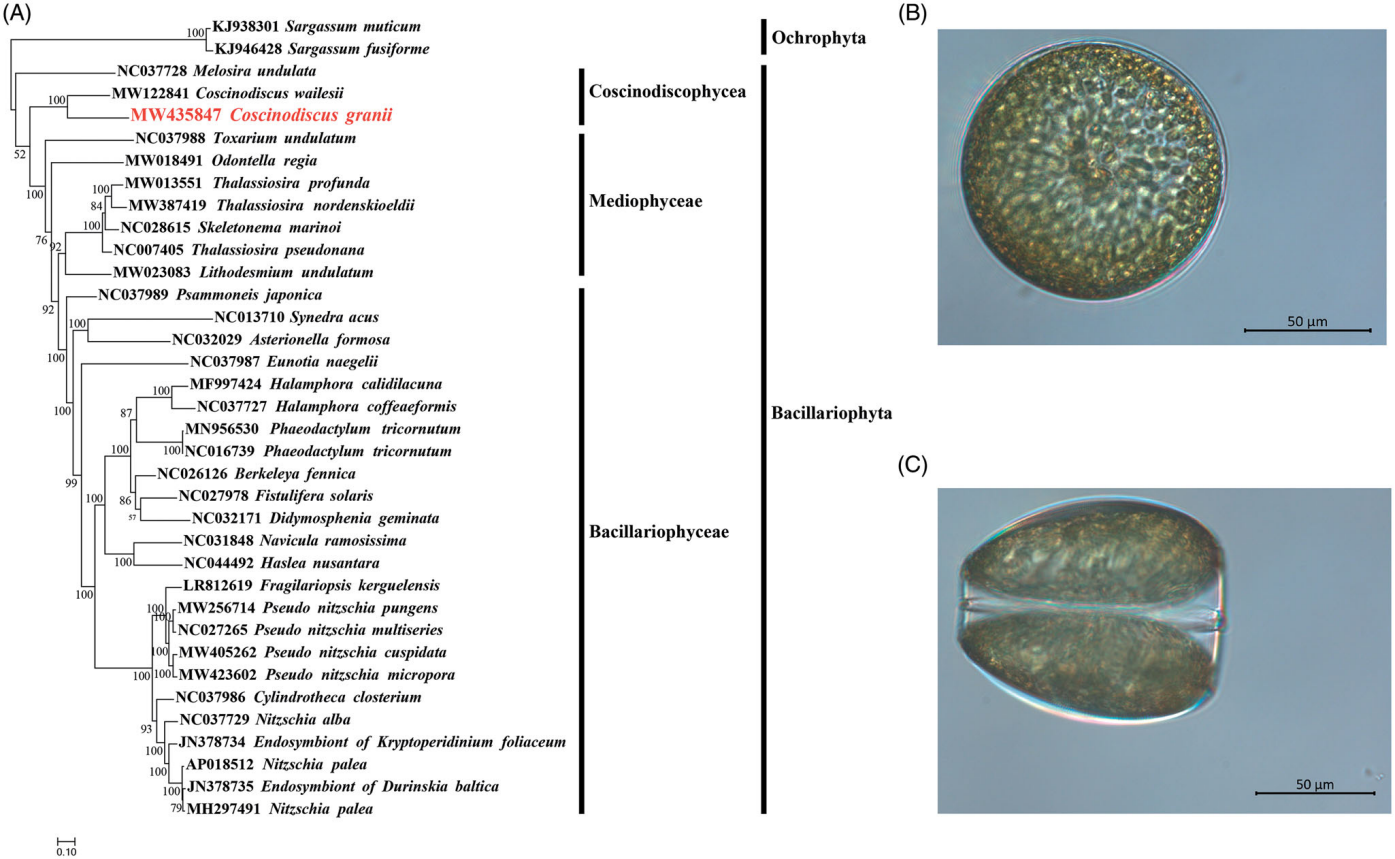
Maximum likelihood (ML) phylogenetic tree using concatenated amino acid sequences of 27 common protein-coding genes (*atp6, 8, 9; cob; cox1, 2, 3*; *nad1-7, 4L, 9*; *rpl2, 13, 14, 19*; *rps3, 4, 8*; and *tatC*) from 34 publicly diatom mitochondrial genomes. Two species of Ochrophyta, *Sargassum fusiforme* (KJ946428) and *Sargassum muticum* (KJ938301), were used as out-group taxa. The numbers beside branch nodes are the percentage of 1000 bootstrap values.

The total DNA of *C. granii* was extracted by using DNAsecure Plant Kit (Tiangen Biotech, Beijing, China). The DNA library was prepared by using the NEB Next^®^ Ultra™ DNA Library Prep Kit for Illumina (NEB, USA). The PCR products were purified using AMPure XP system (Beckman Coulter, Beverly, USA), and libraries were analyzed for size distribution by NGS3K/Caliper and quantified using real-time PCR (Qubit^®^3.0 Flurometer, Invitrogen, USA). After qualification, the library was sequenced using a 2 × 150 bp Illumina NovaSeq 6000 platform (Illumina, USA). Paired-end reads of *C. granii* were assembled using SPAdes (Bankevich et al. 2012). Scaffolds of target mtDNAs were selected from the assembly results using BLASTN (Altschul et al. 1990). The mtDNA sequence was examined using DOTTER (Sonnhammer and Durbin 1995) and validated using the MEM algorithm of BWA (Li and Durbin 2010). The annotation of PCGs, tRNA genes, rRNA genes, and *orf*s was conducted using Open Reading Frame Finder (ORF finder) with SmartBLAST and BLASTP, tRNAscan-SE (Chan and Lowe 2019) and MFannot. The circular mtDNA of *C. granii* was 34,970 bp in size (GenBank accession number: MW435847). The mtDNA encoded a set of 60 genes, including 32 PCGs, two rRNA genes, 24 tRNA genes, and two *orf*s. Genes *rps2*, *rps10*, and *rpl5* that are found in mtDNAs of many other diatom species (Wang et al. 2021) were absent from the mtDNA of *C. granii*. The two *orf*s identified in *C. granii* shared no similarity with these three genes. The GC content of the mtDNA of *C. granii* was 24.30%, which was essentially the same as that of *C. wailesii* (25.00%), and substantially higher than that of *M. undulata* (21.60%) (Pogoda et al. 2019). The 32 PCGs included *atp6*,*8*,*9*; *cob*; *cox1-3*; *nad1-7*,*4L*,*9*,*11*; *rpl2*,*6*,*14*,*16*; *rps3*,*4*,*7*,*8*,*11-14*,*19*; and *tatA*,*C.* The termination codons of most PCGs were TAA (26 of 32 genes) or TAG (6 of 32 genes). Three pairs of overlapping regions were found in the *C. granii* mtDNA, including *rps12*-*rps7* (25 bp), *nad1*-*tatC* (26 bp), and *rp12*-*rps19* (45 bp).

To build the maximum likelihood (ML) phylogenetic tree (Figure 1(A)), all of the 27 shared PCGs from 34 publicly available diatom mtDNAs, were first individually aligned using MAFFT (Katoh and Standley 2013) and then trimmed using trimAL (Capella-Gutierrez et al. 2009) with default parameters: gt = 1, and all amino acid sequences were concatenated using Phyutility (Smith and Dunn 2008). The phylogenetic tree was constructed using IQ-TREE webserver with 1000 bootstrap replications (Trifinopoulos et al. 2016). Two species of Ochrophyta, *Sargassum fusiforme* (KJ946428) and *Sargassum muticum* (KJ938301), were used as out-group taxa. The phylogenetic tree showed that the mtDNAs of species in Bacillariophyceae formed a single clade. The mtDNAs of Mediophyceae and Coscinodiscophyceae each formed a paraphyletic assemblage (Figure 1(A)). *Coscinodiscus granii* (MW435847) was found sister to *C. wailesii* (Huang et al. 2021) in a highly supported clade (Figure 1(A)). Comparative analysis of mtDNAs revealed many rearrangement events. While the order of genes in the mtDNAs of *C. granii* and *C. wailesii* was generally similar, except for the rearrangements of a few gene blocks, including *trnP*(ugg)-*trnY*(gua)-*rps11*, extensive genome rearrangement events were found among mtDNAs of two *Coscinodiscus* species and that of *M. undulata*, including translocation and inversion events. These results suggested that mtDNAs of many more species of the class Coscinodiscophyceae are needed for in-depth understanding of their evolutionary dynamics.

## Data Availability

The genome sequence data that support the findings of this study are openly available in GenBank of NCBI at https://www.ncbi.nlm.nih.gov/ under the accession number MW435847. The associated BioProject, SRA and Bio-Sample numbers are PRJNA689860, SRR13363725 and SAMN17220988, respectively.
